# Community Structure and Functional Gene Profile of Bacteria on Healthy and Diseased Thalli of the Red Seaweed *Delisea pulchra*

**DOI:** 10.1371/journal.pone.0050854

**Published:** 2012-12-03

**Authors:** Neil Fernandes, Peter Steinberg, Doug Rusch, Staffan Kjelleberg, Torsten Thomas

**Affiliations:** 1 The Centre for Marine Bio-Innovation, University of New South Wales, Sydney, Australia; 2 School of Biotechnology and Biomolecular Sciences, University of New South Wales, Sydney, Australia; 3 School of Biological, Earth and Environmental Sciences, University of New South Wales, Sydney, Australia; 4 Advanced Environmental Biotechnology Centre, Nanyang Technological University, Singapore, Singapore; 5 J. Craig Venter Institute, Rockville, Maryland, United States of America; 6 Singapore Centre on Environmental Life Sciences Engineering, Nanyang Technological University, Singapore, Singapore; Laurentian University, Canada

## Abstract

Disease is increasingly viewed as a major factor in the ecology of marine communities and its impact appears to be increasing with environmental change, such as global warming. The temperate macroalga *Delisea pulchra* bleaches in Southeast Australia during warm summer periods, a phenomenon which previous studies have indicated is caused by a temperature induced bacterial disease. In order to better understand the ecology of this disease, the bacterial communities associated with threes type of samples was investigated using 16S rRNA gene and environmental shotgun sequencing: 1) unbleached (healthy) *D. pulchra* 2) bleached parts of *D. pulchra* and 3) apparently healthy tissue adjacent to bleached regions. Phylogenetic differences between healthy and bleached communities mainly reflected relative changes in the taxa *Colwelliaceae*, *Rhodobacteraceae*, *Thalassomonas* and *Parvularcula*. Comparative metagenomics showed clear difference in the communities of healthy and diseased *D. pulchra* as reflected by changes in functions associated with transcriptional regulation, cation/multidrug efflux and non-ribosomal peptide synthesis. Importantly, the phylogenetic and functional composition of apparently healthy tissue adjacent to bleached sections of the thalli indicated that changes in the microbial communities already occur in the absence of visible tissue damage. This shift in unbleached sections might be due to the decrease in furanones, algal metabolites which are antagonists of bacterial quorum sensing. This study reveals the complex shift in the community composition associated with bleaching of *Delisea pulchra* and together with previous studies is consistent with a model in which elevated temperatures reduce levels of chemical defenses in stressed thalli, leading to colonization or proliferation by opportunistic pathogens or scavengers.

## Introduction

Disease in natural communities is increasingly seen as a major ecological factor. Moreover, a number of studies have argued that the frequency and impact of disease on natural communities is on the rise, likely due to the increasing impact of environmental stressors, such as global warming or other anthropogenic effects [1], [2]. The impact of disease is arguably felt most strongly when the affected hosts are biogenic habitat formers, or so-called “ecosystem engineers”, because decline in these organisms results in a fundamental change in the physical structure of the habitat, and the loss of not just the hosts, but of the substantial biodiversity associated with habitat forming species.

In marine systems, to date the most prominent example of disease impacting habitat-forming organisms are tropical reef-building corals [3], [4]. However, on temperate and boreal rocky shorelines, macroalgae (i.e. kelps and other seaweeds) dominate, and there they form the basis for extensive and highly diverse communities [5]. There is now evidence that these macroalgal forests are in decline globally, and one suggested mechanism is that of an increased impact of disease [6].

The red macroalga *Delisea pulchra* [7] is common and in places dominant in the shallow sub-tidal zone in southern Australia, New Zealand, Japan and Antarctica [8]. We have recently described a bleaching phenomenon in this alga, in which portions of the thallus lose their pigmentation and decay [9], [10]. The consequences of this bleaching disease are severe; because of the pattern of bleaching on the thallus relative to the production of reproductive tissue, bleached *D. pulchra* are essentially neutered, with the amount of reproductive tissue an order of magnitude less than that of healthy individuals [11]. Bleaching is most common in summer, but rather than being a direct effect of light or temperature or other environmental stressors, it appears to be due to bacterial infection of (in particular) temperature stressed plants [10], [11]. Two bacteria from the Rosebacter clade, namely *Ruegeria* sp. R11 and *Phaeobacter* sp. LSS9, have been identified from the surface of *D. pulchra*, which can cause bleaching in the laboratory [11], [12], and strain R11 has also been shown experimentally to cause bleaching in the field [11].

Further evidence for the role of bacteria in causing bleaching comes relates to the alga’s antibacterial chemical defenses. *D. pulchra* produces halogenated furanones at its surface [13], [14]. These compounds are strong antibacterials [14]–[20] and one important mechanism for their antibacterial activity is the inhibition of N-acyl homoserine lactone (AHL) based quorum sensing (QS) [13], [16] **.** Furanones in *D. pulchra* are typically at their lowest in summer [21], corresponding to the peak in the incidence of bleaching. A decrease in the furanone concentration of the thallus is also correlated with tissue bleaching [9]. Most compellingly, direct experimental manipulation of furanones results in rapid bleaching of *D. pulchra* in the laboratory [10], [11] and in the field [11].

A number of significant questions remain about the nature of this bacteria-alga interaction, but a critical one is: What is the nature of changes in the microbial community associated with bleaching? To address these questions we performed an in-depth microbial community analysis using 16S rRNA gene sequencing and metagenomics, expanding upon previous community studies based on relatively low-resolution techniques (tRFLP), which showed consistent differences in the community composition of healthy and bleached *D. pulchra* across sampling years, location and depth [9]. We compared bacterial communities from unbleached algae, from bleached tissue, and from apparently healthy (pigmented) tissue adjacent to bleached tissue. This later category is of particular interest, because such tissue, although visibly unaffected, contains furanone levels comparable to bleached tissue [9]. Thus by understanding the microbial communities on chemically poorly defended, but apparently otherwise undamaged tissue, we may gain insight into the progression of the infection process.

## Materials and Methods

### Samples and Community DNA Isolation

Triplicate samples of healthy and bleached individuals of *D. pulchra* were collected at depths of 2–4 m from Bare Island (33°59′38″S, 151°14′00E″), off the coast of Sydney, Australia, during the late Australian summer (25^th^ March 2008). While this sampling in this study is confined to one time and place, we note that the goal here was a more detailed examination of shifts of microbial community composition on *D. pulchra* associated with bleaching, which have already been demonstrated to occur across multiple years and locations [9]. The sampling was covered by permit no P00/0054-6.0 of the New South Wales Department of Industry and Investment. The algae were transported to the laboratory in polyethylene bags containing seawater kept cool by ice packs. Within two hours of collection**,** tissue samples were individually excised from three healthy (H) and three bleached (B) algae, as well as three tissue samples adjacent (A) to bleached tissue of the same thallus (Figure S1). Community DNA from these fresh tissue samples was extracted by a modified enzymatic-detergent lysis protocol [22] (see supplementary information for details).

### Phylogenetic Analysis

A modification of the nested PCR-denaturing gradient gel electrophoresis (DGGE) approach [23] was used to obtain a community fingerprint of all replicate DNA extracted from samples types H, B and A. Further details of the community fingerprint experiments are given in the supplementary information. Clone libraries for the 16S rRNA gene were constructed as previously described [24]. Briefly, 16S rRNA genes were amplified separately from DNA extracted from duplicates of each sample type using PCR primers 27F and 1492R [25]. PCR amplicons were cloned into the Zero blunt TOPO PCR cloning kit (Invitrogen, Carlsbad, Ca, USA) following the manufacturer’s instructions and libraries were sequenced. Forward and reverse reads were assembled and 16S rRNA genes greater 1200 bp were aligned with the SILVA web aligner tool using default parameters [26]. Putative chimeric sequences were identified and removed using the program Mallard [27]. A total of 4670 aligned sequences were clustered by the program MOTHUR [28] into operational taxonomic units (OTUs) using the furthest neighbor method and identity cut-offs of 0.03 representing approximately species-level clustering. In order to identify discriminating species from 16S rRNA gene analysis, OTU-abundance tables were subjected to multivariate statistical analysis using the Similarity Percentage Analysis (SIMPER) [29] implemented in the PRIMER software package (Plymouth Routines in Multivariate Ecological Research) [30]. Bray-Curtis similarity matrices were used to cluster sample types and SIMPER analysis was used to determine the OTUs that contribute to greater than 1% of the total difference between samples from bleached, adjacent and healthy tissues. Because Bray-Curtis similarity measures the contribution of each species, the average dissimilarity between different samples can be expressed in terms of the average contribution from each species. The Ribosomal Database Project (RDP) classifier [31] was then used with default parameters to classify all sequences.

### Metagenomic Analysis

Metagenomic clone libraries were produced and sequenced from algal community DNA using previously described procedures [32], [33]. Briefly, large-scale sequencing was performed on ABI3730XL sequencers for duplicate samples of sample types H, B and A, which corresponded to the ones used for preparation of the 16S rRNA gene libraries. Shotgun sequences were assembled using the Celera Assembler software version 5.1 with twelve percentage errors permitted in the unitigger (utgErrorRate) and 14% error allowed in the overlapper (ovlErrorRate), consensus (cnsErrorRate), and scaffolder (cgwErrorRate) modules. Seed length (merSizeOvl) was set to 14. Overlap trimming, extended clear ranges, and surrogates were turned on. Fragment correction and bubble popping were turned off. In order to filter out eukaryotic DNA contamination, arising from co-extraction of algal DNA, the assembled data was filtered by searching them against The National Center for Biotechnology Information (NCBI) nucleotide database (NT) using BLASTN [34]. Further information on the filtering and the annotation of the metagenomic sequences can be found in the supplementary information. Annotated matches to the COG databases [35] were organized into COG categories. Matrices of raw counts of COGS were standardized to account for the unequal sequence coverage between samples. Bray-Curtis similarity matrices were used to cluster sample types and SIMPER was employed to determine the contribution of each COG to differences between the sample types. Shotgun and 16S rRNA gene sequencing data are available through the Community Cyberinfrastructure for Advanced Microbial Ecology Research and Analysis (accession: CAM_PROJ_BotanyBay).

## Results

### Taxonomic Composition and Differences of Bacterial Communities

**Figure 1:**
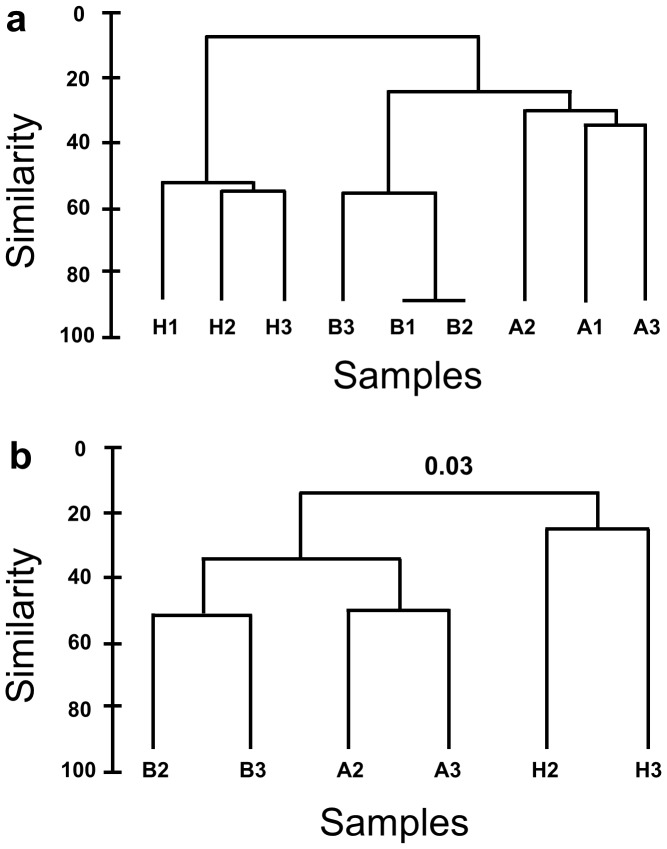
a) Dendogram generated from digitalized bands of the 16S rRNA gene DGGE profile of health *D. pulchra* (H1, 2 and 3), bleached tissue (B1, 2 and 3) and tissue adjacent to bleached tissue (A1, 2 and 3). b) Dendrograms comparing the level of similarity between 16S rRNA gene libraries constructed at 0.03 difference. Sample IDs are the same as for (a).

To confirm that the samples collected in this study show similar patterns of bacterial community shift as seen previously [9], we performed finger-printing analysis (DGGE). Clear differences in the healthy (H) versus bleached (B) samples could be visualized on the DGGE gels (Figure S2) and were reflected in the sample dendograms **(** Figure 1a **)**. Also, the community associated with healthy tissue adjacent to diseased parts of the thalli showed similarities to both the healthy and bleached samples, consistent with previous observations [9].

Filtered and quality-checked 16S rRNA gene libraries (see Material and Methods) consisted of 694 and 1091 sequences for the two healthy tissues of *D. pulchra*, 767 and 812 sequences from bleached tissues and 416 and 890 sequences from tissues extracted from the region adjacent to bleached tissue (Table 1). Chao1-estimates at 0.03 sequence differences showed lower community richness for healthy samples (216 to 151), than for adjacent samples (507 to 937) or bleached thalli (846 and 937) (Table 1). This shows that healthy *D. pulchra* supports less diverse communities than bleached samples (p = 0.0062 for H versus B; unpaired student t-test), which is also reflected in the rarefaction curve analysis (see Figure S3).

**Table 1:** The number of sequences analyzed, OTUs at a cut-off of 0.03, and Chao 1 OTU richness estimates for each 16S rRNA library prepared from bleached tissue (B) healthy tissue (H) and adjacent tissue (A).

Sample	Sequences	OTUs at 0.3	Chao I
H2	694	133	216
H3	1091	101	151
A2	416	234	507
A3	890	392	937
B2	812	364	937
B3	767	284	846

Bray-Curtis similarity analysis and clustering of 16S rRNA gene libraries reflected the patterns seen in the DGGE fingerprinting (Figure 1b). At OTU cut-offs of 0.03 dissimilarity, healthy samples showed less than 20% similarity with the other two samples types, while bleached and adjacent samples shared at least 35% of the sequences found. Adjacent and bleached samples shared as many as 143 sequences, while each of these samples shared only around 30 sequences each with the community of healthy samples (see Figure S4).

**Figure 2:**
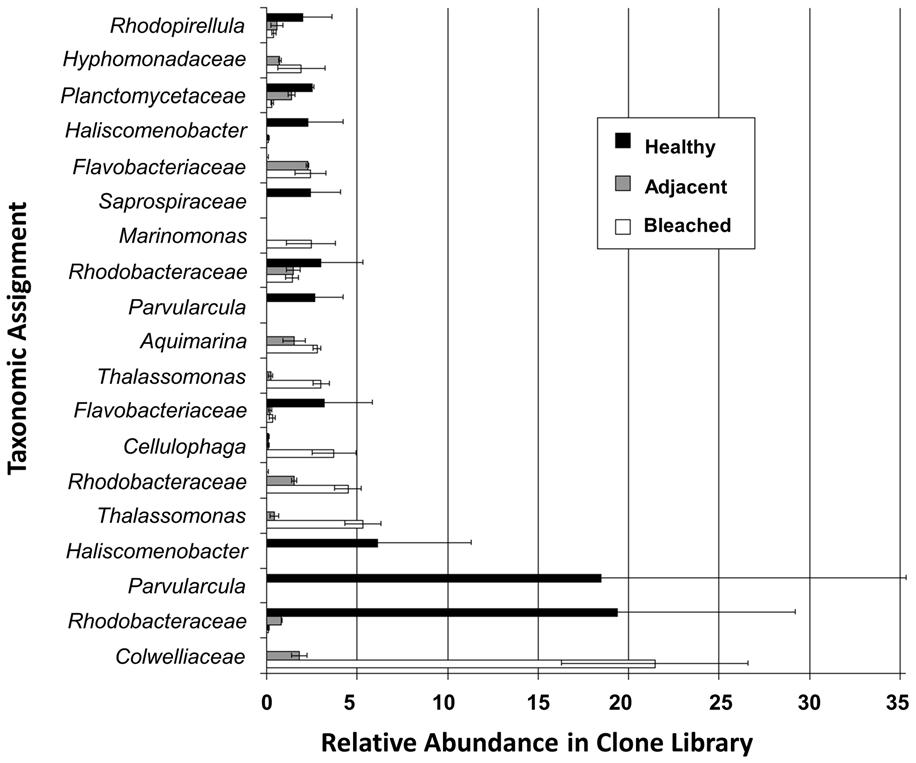
Taxonomic assignments of 16S rRNA gene sequence obtained from bleached tissue, tissue adjacent to bleached tissue and healthy tissue from *Delisea pulchra.* Lowest taxa with RDP classifier confidence level of 90% or greater are shown. Error bars represent the standard error of the mean for two replicates.

Sample discrimination analysis (SIMPER) showed that at the 0.03 difference level only 19 sequences accounted for 60% of the average dissimilarity between bleached and healthy samples (see Figure S5). The taxonomic assignment of these “discriminatory” OTUs and their relative abundance in the 16S rRNA gene clone libraries of all three sample types are given in Figure 2. An OTU belonging to the family *Colwelliaceae* had an abundance of 21.46% in bleached samples and was reduced to an abundance of about 2% in adjacent tissue, and was not detected in healthy thalli. Similar trends were observed for OTUs belonging to the taxa *Thalassomonas*, *Rhodobacteraceae*, *Cellulophaga*, *Aquimarina, Marinomonas* and *Hypomonadaceae.* In contrast, healthy samples had a very low abundance in OTUs belonging to the *Rhodobacteraceae, Parvularcula*, *Haliscomenobacter*, *Flavobacteriaceae* and *Saprospiraceae* compared to the other two sample types. We also found no evidence for the presence or enrichment of *Ruegeria* sp. R11 and *Phaeobacter* sp. LSS9 on bleached samples.

Together this data show clear differences in the taxonomic composition of communities of healthy *D. pulchra* versus thalli that are bleached or parts that appear healthy and are located immediately adjacent to bleached regions. These changes seem to be largely driven by substantial shifts in the abundance of a few OTUs. To better understand these changes, we further analysed these bacterial communities using a metagenome sequencing approach.

### Functional Gene Profiles of Healthy, Bleached and Adjacent Communities

**Figure 3:**
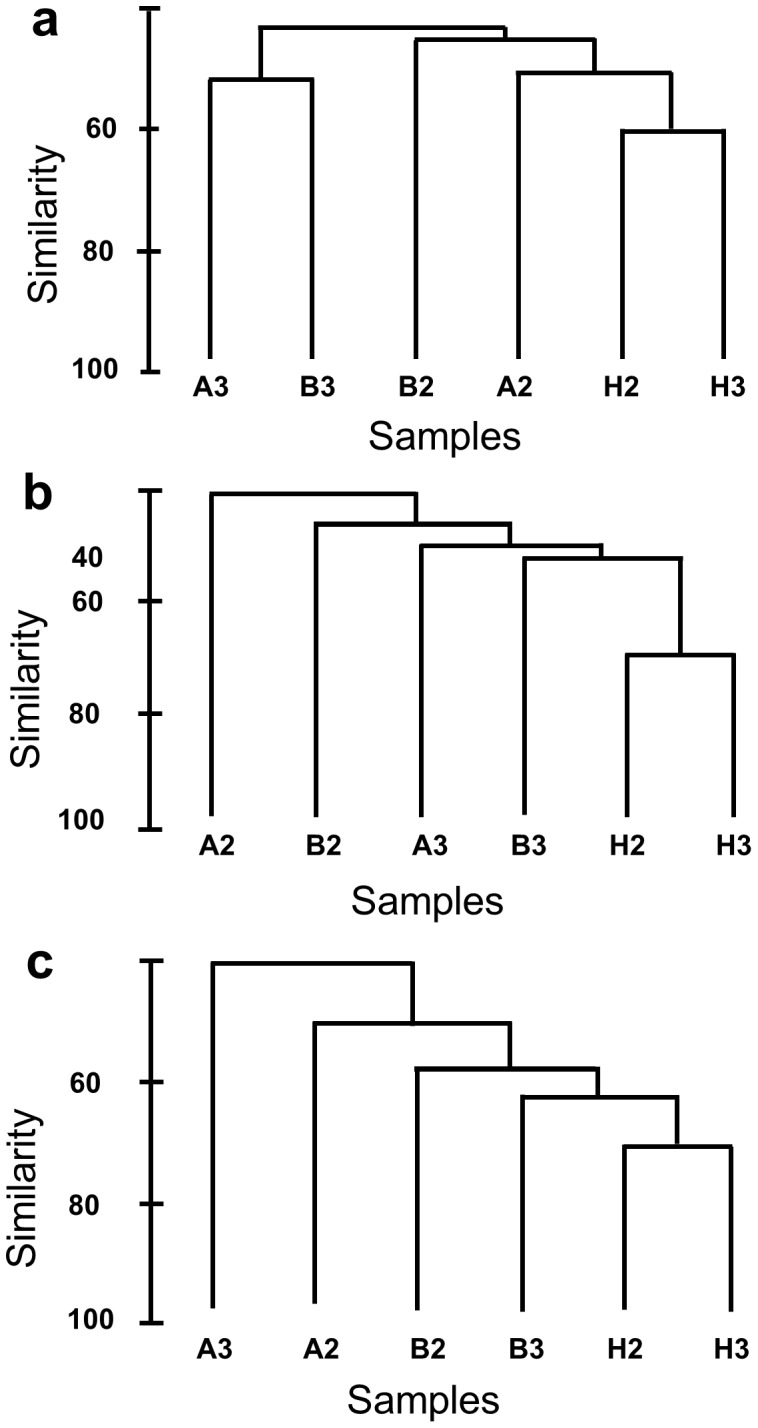
Dendrograms comparing the level of similarity between metagenomic libraries constructed from bleached tissue (B), from tissue adjacent to bleached tissue (A) and healthy tissue (H) using a matrix containing ORFs that could be matched to COGs with E-value cut-offs of 10 ^−**5**^ **(a), 10** ^−**10**^ **(b) and 10** ^−**20**^ **(b).**

Shotgun sequencing, functional annotation and statistical analysis of six microbial metagenomes from two samples each of diseased, healthy and adjacent tissues of *D. pulchra* identified a number of “discriminatory” functions and those were used to cluster samples in dendograms (Table 2, Figure 3) and to ordinate by multi-dimensional scaling (MDS) (Figure S6). Overall, functional profiles based in COG annotation from healthy samples clustered together and were distinct from the bleached and adjacent samples. This clustering based on functional profile reflected those seen for the taxonomic assignment.

**Table 2:** Number of open reading frames (ORFs), number of ORFs assigned to COGs and the percentage of total ORFs assigned to COGs (cut-off of 10^−20^) for metagenomic libraries prepared from bleached tissue (B) healthy tissue (H) and adjacent tissue (A).

Sample	Number of ORFs	ORFs assigned to COGs	% of ORFs assigned to COGs
H2	55724	18205	33%
H3	44235	19876	45%
A2	6998	1542	22%
A3	7613	1959	26%
B2	59767	12972	22%
B3	71813	21521	30%

**Figure 4:**
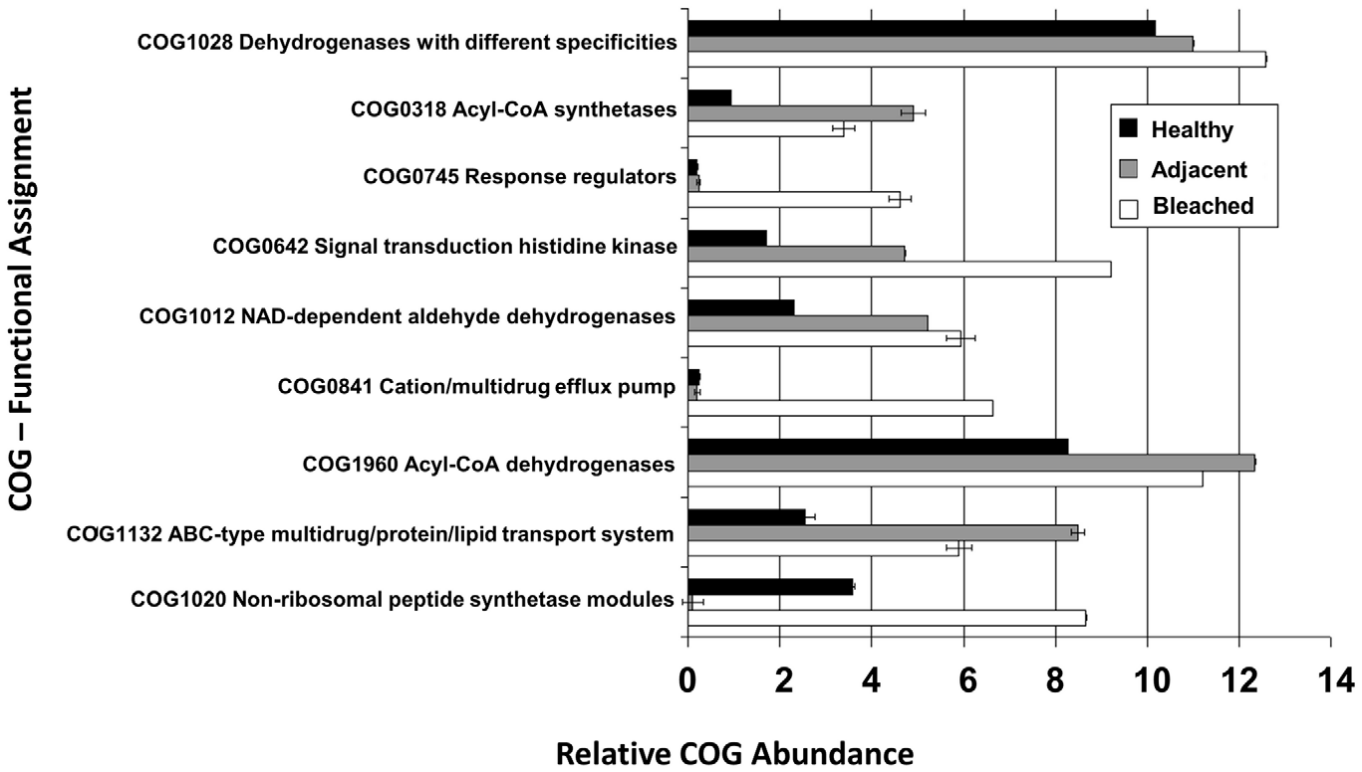
Relative abundance of COGs in metagenomic libraries prepared from bleached tissue, tissue adjacent to bleached tissue and healthy tissue from *Delisea pulchra.* Error bars represent the standard error of the mean for two replicates.

A ranked plot of the relative contribution of each COG function showed that only nine COG functions make up for 60% of the average dissimilarity between bleached and healthy samples (Figure S7). The relative abundances of those discriminatory functions in the three different samples are shown in Figure 4. In general, the individual discriminatory functions became more abundant in sample types A and B versus healthy sample. Substantial enrichment in sample types A and B was observed for the functions acyl-CoA synthetases (AMP-forming)/AMP-acid ligases II (COG318), response regulators consisting of a CheY-like receiver domain and a winged-helix DNA-binding domain (COG745), signal transduction histidine kinase (COG0642), NAD-dependent aldehyde dehydrogenases (COG1012), cation/multidrug efflux pump (COG841), ABC-type multidrug/protein/lipid transport system (COG 1132) and non-ribosomal peptide synthetase modules and related proteins (COG1020).

## Discussion

### Bacterial Community of Bleached and Adjacent Tissue

Bleaching of *D. pulchra* is associated with clear changes in the community composition, not only directly at the affected part of the thallus, but also in the surrounding areas. Community richness of bleached and adjacent tissue was in general similar, but greatly increased (>3-fold; see Table 1) compared to healthy samples. This increased diversity could be the result of changes in the furanone concentrations, which are high in healthy *D. pulchra* [19], but greatly reduced in bleached algae and their apparently healthy tissue [9]. The loss of selective and inhibitory effect of the alga’s chemical defense could thus allow for a greater variety of bacteria to colonize and these might potentially include opportunistic pathogens and scavengers (see below). Increases in bacterial diversity have also been observed when the Caribbean coral *Montastraea faveolata* was afflicted by White Plague Disease [36] and when the common reef-building coral, *Porites cylindrica* was exposed to stressful environmental events [37].

Communities of diseased thalli are characterized by an abundance of OTUs belonging to the genera *Cellulophaga* and *Thalassomonas* as well as the family *Colwelliaceae*, some of which are also found in adjacent tissue, but absent on healthy algae. Algal diseases are often associated with cell-wall degradation and members of the genus *Cellulophaga* have been shown to exhibit potent extra-cellular alginolytic or agarolytic activity [38]. OTUs within the *Colwelliaceae* account for most of the difference between healthy and bleached tissue and this family contains several genera with known or putative pathogenic and anti-algal representatives. For example, *Thalassomona loyana* has been implicated as a pathogen for ‘white plague-like disease’ in corals in the Red Sea [39]. Bacteria within the genera *Alteromonas* and *Pseudoalteromonas* (family *Colwelliaceae*) have also been shown to exhibit algicidal activity against eukaryotic microalgae [40] and have been found associated with decaying matter of algal blooms [41].

These observations are consistent with two alternative models for the changes in microbial communities associated with bleaching in *D. pulchra.* Firstly, changes could be a result of opportunistic pathogens that either colonise or proliferate on relatively chemically undefended tissue and subsequently cause the bleaching disease. The absence of previously identified pathogens of *D. pulchra* (i.e. *Ruegeria* sp. R11 and *Phaeobacter* sp. LSS9) in the samples taken in the current study would also indicate that other bacterial taxa might have the ability to induce disease. Secondly, an alternative model would be that disease occurs independently of pathogens and that some of the taxa enriched on bleached thallus might be involved in tissue decay or nutrient scavenging post disease initiation. These taxa would hence be considered to be opportunistic, secondary colonizers. Further time-series experiments under controlled conditions and tracking of potential pathogens identified here (e.g. *Thalassomona*) and in our previous work [11], [12] across a larger number of disease events are required to distinguish between these possibilities.

### Loss of Microbiota from Healthy Samples

Taxonomically, healthy tissue was characterized by the abundance of bacteria belonging to the genera *Parvularcula* and *Haliscomenobacter,* and the family *Rhodobacteraceae*. The most abundant *Rhodobacteraceae* phylotype (19% of the sequences found) could also be assigned with moderate confidence (70%) to the genus *Thioclava.* The genus *Thioclava* as well as the second most abundant group, the genus *Parvularcula*, harbor genes for the degradation of the abundant algal osmolyte dimethylsulfoniopropionate (DMSP) [42] and are known to oxidize inorganic sulfur metabolites [43], such as the byproducts of DMSP degradation [44]. Bacteria involved in the biogeochemical cycling of sulfur are also abundant in coral-associated communities and are thought to play a significant role in structuring the community and to be important for coral health [45], [46]. Members of the family *Saprospiraceae* (order *Bacteroidetes*), to which the genus *Haliscomenobacter* also belongs (see Figure 2), have also been isolated from marine sponges and algae from the southern coastline of Thailand [47]. While little is known about the ecophysiology of other *Saprospiraceae*, members of the *Bacteroidetes* are generally associated with the degradation of complex organic materials and *Haliscomenobacter hydrossis* is abundant in activated sludge flocs and thought to be involved in the hydrolysis of polysaccharides [48].

While the ecological role of these bacterial associates that dominate the community of healthy *D. pulchra* is not clear, they belong to taxonomic groups that are commonly found on marine algae [49]–[52]. These groups might thus represent epiphytes that are generally not harmful to the host or are important for structuring a stable microbial community. Importantly, these community members are also in lower abundance on bleached tissue as well as on adjacent parts, where there are no visible changes. This observation would be consistent with a probiotic model, which was proposed for corals [53] and posits that the normal microbiota associated with living surfaces offers protection against microbial infection and disease. According to this concept, the community of the adjacent tissue would have already lost key members of the “normal” microbiota, a shift that may allow for subsequent proliferation of harmful bacteria in the community. This community shift might be due to the loss of algal defense (furanone) and could result in a dysbiosis of the epiphytic bacterial community prior to disease development. Thus it might be important to consider the state of the microbial community prior to disease development and define its effect on disease initiation or outcome.

### Taxonomic Shifts are Accompanied by Changes in the Functional Gene Composition

Accompanying the taxonomic changes, shotgun metagenomic analysis revealed clear differences also in the functional gene composition across communities from healthy, diseased and adjacent tissue. In particular, a series of functional genes were enriched in communities of diseased tissue. These include non-ribosomal peptide synthetases (NRPSs; COG1020), which synthesize molecules with a range of biological functions [54], including antibiotics [55], siderophores [56] and other virulence determinants [57] [58], [59]. Either of these functions could allow bacteria on diseased *D. pulchra* to outcompete other epiphytes, support growth or act directly as a virulence factor in the bleaching disease.

Abundant in communities of diseased tissue were also cation/multidrug efflux pumps (COG0841), which belong to a larger group of multidrug resistance pumps (MDRs) that bacteria utilize to extrude toxins [60]. Recently, the MDRs of plant pathogenic bacteria, including *Erwinia amylovora*, *Dickeya* spp. and *Agrobacterium tumefaciens*, have been shown to play a crucial role in the resistance to plant antimicrobials and their inactivation compromised initial colonization and virulence [61]. MDRs are also important for bacterial competition and colonization within surface-associated bacterial communities. For example, efﬂux pump mutants of the plant pathogens *E. chrysanthemi* and *E. amylovora* are less infectious than the wild-type strain when co-inoculated with other endophytic bacteria producing antimicrobial compounds [62], [63].

Two-component signal transduction (TCST) systems (COG 0642) are used by many bacteria to sense environmental signals and modulate expression of genes [64], including ones involved in virulence, antibiotic resistance responses, and colonization in many pathogenic bacteria [65], [66]. For example, in the plant pathogenic bacteria *Xanthomonas campestris*, *Pectobacterium carotovorum* and *Ralstonia solanacearum*, TCST systems have been shown to provide environmental signal inputs to global virulence regulation [67]. The abundance of TCST systems found in communities of bleached tissue could thus enable bacteria to modulate gene expression in response to environmental changes and regulate virulence genes responsible for bleaching. The potential importance of regulation in disease was also recently highlighted in the analysis of the genomes for the *D. pulchra* pathogens *Nautella* sp. R11 and *Phaeobacter* sp. LSS9 [12]. These two pathogens contained no unique set of putative virulence factors when compared to other closely related non-pathogenic strains, but had a unique LuxR-type regulator that was hypothesized to regulated virulence.

### Conclusion

Marine macroalgae, including *D. pulchra*, are major habitat formers in temperate ecosystems, and diseases of these organisms have the potential to cause significant impacts on community structure, population levels and biodiversity in marine ecosystems [1]. Campbell et al [9] have proposed a model for bleaching in *D. pulchra*, in which environmental stress (particularly elevated seawater temperatures) leads to a decrease in chemical defenses (furanones), which in turn leads to a shift in bacterial community composition, which can include opportunistic pathogens or scavangers. This shift might be further exacerbated by the loss of certain key members of the “normal” microbiota of *D. pulchra,* which might have probiotic functions. Our observations are consistent with such processes and we have identified bacterial species that might be involved in either the disease initiation or secondary scavenging. Functional gene analysis of communities of bleached sample revealed further an abundance of activities that are likely to play key roles in algal colonization and competiton. However the role of these groups of bacteria and genes in the bleaching disease of *D. pulchra* needs further experimental confirmation. Further advances in understanding the bleaching disease in *D. pulchra* would require tracking temporal changes in key species, and in the expression of these key functional genes, at different stages of disease.

## Supporting Information

Figure S1 Bleached *Delisea pulchra* collected from Bare Island, Sydney, Australia. The bleached section in the mid thallus region is indicated by the arrowhead.
(TIF)

Figure S2 Comparison of different samples by DGGE fingerprinting. Lane M: DGGE Markers, Lanes A1, A2 and A3 contain DGGE bands from tissue adjacent to bleached tissue, Lanes B1, B2 and B3, DGGE bands from bleached tissue, Lanes H1, H2 and H3, DGGE bands from healthy tissue.
(TIF)

Figure S3 Rarefaction curves comparing the number of OTUs at a distance of 0.03 between 16S rRNA gene libraries constructed bleached tissue (B), from tissue adjacent to bleached tissue (A) and healthy tissue (H).
(TIF)

Figure S4 Venn diagrams showing the number of OTUs shared between communities on bleached tissue (B) healthy tissue (H) and adjacent tissue (A) with OTUs at 0.03 difference.
(TIF)

Figure S5 The contribution of different OTUs (at 0.03 sequence difference cut-off) to difference between 16S rRNA genes libraries from bleached and healthy samples.
(TIF)

Figure S6 Multidimensional-scaling (MDS) plots comparing the level of similarity between metagenomic libraries constructed from bleached tissue (B), from tissue adjacent to bleached tissue (A) and healthy tissue (H) using a matrix containing ORFs that could be matched to COGs at E-value cut-offs smaller 10^−5^ (**1**), 10^−10^ (**2**) and 10^−20^ (**3**).
(TIF)

Figure S7 The contribution of individual COGs to the difference between metagenomic libraries from bleached and healthy samples.
(TIF)

Text S1 Supplementary Material and Methods.
(DOC)

## References

[1] Harvell CD, Mitchell CE, Ward JR, Altizer S, Dobson AP, et al (2002) Climate warming and disease risks for terrestrial and marine biota. Science 296: 2158–2162. 12077394 10.1126/science.1063699

[2] Lafferty KD, Porter JW, Ford SE (2004) Are diseases increasing in the ocean? Annu Rev Ecol Evol Syst 35: 31–54.

[3] Rosenberg E, Koren O, Reshef L, Efrony R, Zilber-Rosenberg I (2007) The role of microorganisms in coral health, disease and evolution. Nat Rev Microbiol 5: 355–362. 17384666 10.1038/nrmicro1635

[4] Weil E, Smith G, Gil-Agudelo DL (2006) Status and progress in coral reef disease research. Dis Aquat Org 69: 1–7. 16703761 10.3354/dao069001

[5] Kerswell AP (2006) Global biodiversity patterns of benthic marine algae. Ecology 87: 2479–2488. 17089657 10.1890/0012-9658(2006)87[2479:gbpobm]2.0.co;2

[6] Steneck RS, Graham MH, Bourque BJ, Corbett D, Erlandson JM, et al (2002) Kelp forest ecosystems: biodiversity, stability, resilience and future. Environ Conserv 29: 436–459.

[7] Montagne C (1844) Quelque observations touchant la structure et la fructification des genres *Ctenodus*, *Delisea*, et Lenormandia de la famille des Floridees. Ann Sci Nat 1: 151–161.

[8] Papenfuss G (1964) Catalogue and bibliography of Antarctic and sub-Antarctic benthic marine algae In: Bibliography of the Antarctic Seas. (Lee, M.O. Eds). Washington D.C.: American Geophysical Union. 1–76 p.

[9] Campbell AH, Harder T, Nielsen S, Kjelleberg S, Steinberg PD (2011) Climate change and disease: bleaching of a chemically defended seaweed. Global Change Biol 17: 2958–2970.

[10] Case RJ, Longford SR, Campbell AH, Low A, Tujula N, et al (2011) Temperature induced bacterial virulence and bleaching disease in a chemically defended marine macroalga. Environ Microbiol 13: 529–537. 20946533 10.1111/j.1462-2920.2010.02356.x

[11] Campbell A (2011) The ecology of bacterially mediated bleaching in a chemically defended seaweed. [PhD Thesis]: The University of New South Wales, Sydney, NSW, Australia.

[12] Fernandes N, Case RJ, Longford SR, Seyedsayamdost MR, Steinberg PD, et al (2011) Genomes and Virulence Factors of Novel Bacterial Pathogens Causing Bleaching Disease in the Marine Red Alga Delisea pulchra. Plos One 6: e27387. 22162749 10.1371/journal.pone.0027387 PMC3230580

[13] De Nys R, Givskov M, Kumar N, Kjelleberg S, Steinberg P (2006) Furanones. Prog Mol Subcell Biol 42: 55–86. 16805438 10.1007/3-540-30016-3_2

[14] De Nys R, Steinberg P, Willemsen P, Dworjanyn S, Gabelish C, et al (1995) Broad spectrum effects of secondary metabolites from the red alga *Delisea pulchra* in antifouling assays. Biofouling 8: 259–271.

[15] Gram L, de Nys R, Maximilien R, Givskov M, Steinberg P, et al (1996) Inhibitory effects of secondary metabolites from the red alga *Delisea pulchra* on swarming motility of *Proteus mirabilis* . Appl Environ Microbiol 62: 4284–4287. 16535454 10.1128/aem.62.11.4284-4287.1996 PMC1388992

[16] Hentzer M, Wu H, Andersen J, Riedel K, Rasmussen T, et al (2003) Attenuation of *Pseudomonas aeruginosa* virulence by quorum sensing inhibitors. EMBO J 22: 3803–3815. 12881415 10.1093/emboj/cdg366 PMC169039

[17] Hentzer M, Riedel K, Rasmussen T, Heydorn A, Andersen J, et al (2002) Inhibition of quorum sensing in *Pseudomonas aeruginosa* biofilm bacteria by a halogenated furanone compound. Microbiology 148: 87–102. 11782502 10.1099/00221287-148-1-87

[18] Manefield M, Welch M, Givskov M, Salmond G, Kjelleberg S (2001) Halogenated furanones from the red alga, *Delisea pulchra*, inhibit carbapenem antibiotic synthesis and exoenzyme virulence factor production in the phytopathogen *Erwinia carotovora* . FEMS Microbiol Lett 205: 131–138. 11728727 10.1111/j.1574-6968.2001.tb10936.x

[19] Maximilien R, de Nys R, Holmström C, Gram L, Givskov M, et al (1998) Chemical mediation of bacterial surface colonisation by secondary metabolites from the red alga *Delisea pulchra* . Aquat Microb Ecol 15: 233–246.

[20] Rogers C, De Nys R, Charlton T, Steinberg P (2000) Dynamics of algal secondary metabolites in two species of sea hare. J Chem Ecol 26: 721–744.

[21] Wright J, De Nys R, Steinberg P (2000) Geographic variation in halogenated furanones from the red alga *Delisea pulchra* and associated herbivores and epiphytes. Mar Ecol Prog Ser 207: 227–241.

[22] Burke C, Kjelleberg S, Thomas T (2009) Selective extraction of bacterial DNA from the surfaces of macroalgae. Appl Environ Microbiol 75: 252–256. 18978081 10.1128/AEM.01630-08 PMC2612226

[23] Muyzer G, Smalla K (1998) Application of denaturing gradient gel electrophoresis (DGGE) and temperature gradient gel electrophoresis (TGGE) in microbial ecology. Antonie van Leeuwenhoek 73: 127–141. 9602286 10.1023/a:1000669317571

[24] Shaw A, Halpern A, Beeson K, Tran B, Venter J, et al (2008) It's all relative: ranking the diversity of aquatic bacterial communities. Environ Microbiol 10: 2200–2210. 18637951 10.1111/j.1462-2920.2008.01626.x

[25] Lane D (1991) 16S/23S rRNA sequencing. In: Nucleic acid techniques in bacterial systematics. (Stackebrandt E & Goodfellow M, eds). J. Wiley & Sons, Chichester. 115–175 p.

[26] Pruesse E, Quast C, Knittel K, Fuchs B, Ludwig W, et al (2007) SILVA: a comprehensive online resource for quality checked and aligned ribosomal RNA sequence data compatible with ARB. Nucleic Acids Research 35: 7188–7196. 17947321 10.1093/nar/gkm864 PMC2175337

[27] Ashelford K, Chuzhanova N, Fry J, Jones A, Weightman A (2006) New screening software shows that most recent large 16S rRNA gene clone libraries contain chimeras. Appl Environ Microbiol 72: 5734–5741. 16957188 10.1128/AEM.00556-06 PMC1563593

[28] Schloss P, Westcott S, Ryabin T, Hall J, Hartmann M, et al (2009) Introducing mothur: open-source, platform-independent, community-supported software for describing and comparing microbial communities. Appl Environ Microbiol 75: 7537–7541. 19801464 10.1128/AEM.01541-09 PMC2786419

[29] Clarke KR (1993) Non-parametric multivariate analyses of changes in community structure. Aust J Ecol 18: 117–143.

[30] Clarke K, Gorley R (2006) PRIMER v6: User Manual/tutorial. Primer-E Ltd Plymouth.

[31] Cole J, Wang Q, Cardenas E, Fish J, Chai B, et al (2008) The Ribosomal Database Project: improved alignments and new tools for rRNA analysis. Nucleic Acids Res 37: 141–145. 10.1093/nar/gkn879 PMC2686447 19004872

[32] Thomas T, Rusch D, DeMaere MZ, Yung PY, Lewis M, et al (2010) Functional genomic signatures of sponge bacteria reveal unique and shared features of symbiosis. ISME J 4: 1557–1567. 20520651 10.1038/ismej.2010.74

[33] Rusch DB, Halpern AL, Sutton G, Heidelberg KB, Williamson S, et al (2007) The Sorcerer II Global Ocean Sampling expedition: Northwest Atlantic through Eastern Tropical Pacific. PLoS Biol 5: 398–431. 10.1371/journal.pbio.0050077 PMC1821060 17355176

[34] Altschul SF, Madden TL, Schaffer AA, Zhang J, Zhang Z, et al (1997) Gapped BLAST and PSI-BLAST: a new generation of protein database search programs. Nucleic Acids Res 25: 3389–3402. 9254694 10.1093/nar/25.17.3389 PMC146917

[35] Tatusov RL, Fedorova ND, Jackson JD, Jacobs AR, Kiryutin B, et al (2003) The COG database: an updated version includes eukaryotes. BMC Bioinformatics 4: 41–54. 12969510 10.1186/1471-2105-4-41 PMC222959

[36] Sunagawa S, DeSantis T, Piceno Y, Brodie E, DeSalvo M, et al (2009) Bacterial diversity and White Plague Disease-associated community changes in the Caribbean coral *Montastraea faveolata* . ISME J 3: 512–521. 19129866 10.1038/ismej.2008.131

[37] Garren M, Raymundo L, Guest J, Harvell CD, Azam F, et al (2009) Resilience of Coral-Associated Bacterial Communities Exposed to Fish Farm Effluent. Plos One 4: e7319. 19806190 10.1371/journal.pone.0007319 PMC2751826

[38] Bowman JP (2000) Description of *Cellulophaga algicola sp. nov.*, isolated from the surfaces of Antarctic algae, and reclassification of *Cytophaga uliginosa* (ZoBell and Upham 1944) Reichenbach 1989 as *Cellulophaga uliginosa comb. nov* . Int J Syst Evol Microbiol 50: 1861–1868. 11034497 10.1099/00207713-50-5-1861

[39] Thompson F, Barash Y, Sawabe T, Sharon G, Swings J, et al (2006) *Thalassomonas loyana* sp. nov., a causative agent of the white plague-like disease of corals on the Eilat coral reef. Int J Syst Evol Microbiol 56: 365–368. 16449441 10.1099/ijs.0.63800-0

[40] Mayali X, Azam F (2004) Algicidal Bacteria in the Sea and their Impact on Algal Blooms. J Eukaryot Microbiol 51: 139–144. 15134248 10.1111/j.1550-7408.2004.tb00538.x

[41] Kelly KM, Chistoserdov AY (2001) Phylogenetic analysis of the succession of bacterial communities in the Great South Bay (Long Island). FEMS Microbiol Ecol 35: 85–95. 11248393 10.1111/j.1574-6941.2001.tb00791.x

[42] Oh HM, Kang I, Vergin KL, Kang D, Rhee KH, et al (2011) Complete Genome Sequence of Strain HTCC2503T of *Parvularcula bermudensis*, the Type Species of the Order“*Parvularculales*” in the Class *Alphaproteobacteria* . J Bacteriol 193: 305–306. 21037002 10.1128/JB.01205-10 PMC3019957

[43] Sorokin DY, Tourova TP, Spiridonova EM, Rainey FA, Muyzer G (2005) *Thioclava pacifica* gen. nov., sp. nov., a novel facultatively autotrophic, marine, sulfur-oxidizing bacterium from a near-shore sulfidic hydrothermal area. Int J Syst Evol Microbiol 55: 1069–1075. 15879235 10.1099/ijs.0.63415-0

[44] Wagner-Döbler I, Biebl H (2006) Environmental biology of the marine Roseobacter lineage. Microbiology 60: 255–280. 10.1146/annurev.micro.60.080805.142115 16719716

[45] Raina JB, Tapiolas D, Willis BL, Bourne DG (2009) Coral-associated bacteria and their role in the biogeochemical cycling of sulfur. Appl Environ Microbiol 75: 3492–3501. 19346350 10.1128/AEM.02567-08 PMC2687302

[46] Raina JB, Dinsdale EA, Willis BL, Bourne DG (2010) Do the organic sulfur compounds DMSP and DMS drive coral microbial associations? Trends Microbiol 18: 101–108. 20045332 10.1016/j.tim.2009.12.002

[47] Hosoya S, Arunpairojana V, Suwannachart C, Kanjana-Opas A, Yokota A (2006) *Aureispira marina* gen. nov., sp. nov., a gliding, arachidonic acid-containing bacterium isolated from the southern coastline of Thailand. Int J Syst Evol Microbiol 56: 2931–2935. 17159001 10.1099/ijs.0.64504-0

[48] Xia Y, Kong Y, Thomsen TR, Halkjaer Nielsen P (2008) Identification and ecophysiological characterization of epiphytic protein-hydrolyzing *saprospiraceae* (“*Candidatus Epiflobacter*” spp.) in activated sludge. Appl Environ Microbiol 74: 2229–2238. 18263744 10.1128/AEM.02502-07 PMC2292613

[49] Burke C, Thomas T, Lewis M, Steinberg P, Kjelleberg S (2010) Composition, uniqueness and variability of the epiphytic bacterial community of the green alga *Ulva australis* . ISME J 5: 590–600. 21048801 10.1038/ismej.2010.164 PMC3105733

[50] Longford SR, Tujula NA, Crocetti GR, Holmes AJ, Holmström C, et al (2007) Comparisons of diversity of bacterial communities associated with three sessile marine eukaryotes. Aquat Microb Ecol 48: 217–229.

[51] Staufenberger T, Thiel V, Wiese J, Imhoff JF (2008) Phylogenetic analysis of bacteria associated with *Laminaria saccharina* . FEMS Microbiol Ecol 64: 65–77. 18328081 10.1111/j.1574-6941.2008.00445.x

[52] Nylund GM, Persson F, Lindegarth M, Cervin G, Hermansson M, et al (2010) The red alga *Bonnemaisonia asparagoides* regulates epiphytic bacterial abundance and community composition by chemical defence. FEMS Microbiol Ecol 71: 84–93. 19878319 10.1111/j.1574-6941.2009.00791.x

[53] Reshef L, Koren O, Loya Y, Zilber Rosenberg I, Rosenberg E (2006) The coral probiotic hypothesis. Environ Microbiol 8: 2068–2073. 17107548 10.1111/j.1462-2920.2006.01148.x

[54] Finking R, Marahiel MA (2004) Biosynthesis of nonribosomal peptides. Microbiology 58: 453–488. 10.1146/annurev.micro.58.030603.123615 15487945

[55] Vining LC (1990) Functions of secondary metabolites. Annual Review of Microbiology 44: 395–427. 10.1146/annurev.mi.44.100190.002143 2252388

[56] Challis GL, Ravel J, Townsend CA (2000) Predictive, structure-based model of amino acid recognition by nonribosomal peptide synthetase adenylation domains. Chem Biol 7: 211–224. 10712928 10.1016/s1074-5521(00)00091-0

[57] Wyatt MA, Wang W, Roux CM, Beasley FC, Heinrichs DE, et al (2010) *Staphylococcus aureus* Nonribosomal Peptide Secondary Metabolites Regulate Virulence. Science 329: 294–296. 20522739 10.1126/science.1188888

[58] Groll M, Schellenberg B, Bachmann AS, Archer CR, Huber R, et al (2008) A plant pathogen virulence factor inhibits the eukaryotic proteasome by a novel mechanism. Nature 452: 755–758. 18401409 10.1038/nature06782

[59] Arrebola E, Cazorla FM, Romero D, Pérez-García A, de Vicente A (2007) A nonribosomal peptide synthetase gene (mgoA) of *Pseudomonas syringae pv. syringae* is involved in mangotoxin biosynthesis and is required for full virulence. Mol Plant Microbe Interact 20: 500–509. 17506328 10.1094/MPMI-20-5-0500

[60] Tegos G, Stermitz FR, Lomovskaya O, Lewis K (2002) Multidrug pump inhibitors uncover remarkable activity of plant antimicrobials. Antimicrob Agents Chemother 46: 3133–3141. 12234835 10.1128/AAC.46.10.3133-3141.2002 PMC128777

[61] Martinez JL, Sánchez MB, Martínez-Solano L, Hernandez A, Garmendia L, et al (2009) Functional role of bacterial multidrug efflux pumps in microbial natural ecosystems. FEMS Microbiol Rev 33: 430–449. 19207745 10.1111/j.1574-6976.2008.00157.x

[62] Burse A, Weingart H, Ullrich MS (2004) NorM, an *Erwinia amylovora* multidrug efflux pump involved in in vitro competition with other epiphytic bacteria. Appl Environ Microbiol 70: 693–703. 14766544 10.1128/AEM.70.2.693-703.2004 PMC348922

[63] Llama-Palacios A, Lopez-Solanilla E, Rodriguez-Palenzuela P (2002) The ybiT gene of *Erwinia chrysanthemi* codes for a putative ABC transporter and is involved in competitiveness against endophytic bacteria during infection. Appl Environ Microbiol 68: 1624–1630. 11916677 10.1128/AEM.68.4.1624-1630.2002 PMC123845

[64] García VE, Sciara M, Castelli M (2010) Two component systems in the spatial program of bacteria. Curr Opin Microbiol 13: 210–218. 20138002 10.1016/j.mib.2009.12.012

[65] Beier D, Gross R (2006) Regulation of bacterial virulence by two-component systems. Curr Opin Microbiol 9: 143–152. 16481212 10.1016/j.mib.2006.01.005

[66] Calva E, Oropeza R (2006) Two-component signal transduction systems, environmental signals, and virulence. Microb Ecol 51: 166–176. 16435167 10.1007/s00248-005-0087-1

[67] Mole BM, Baltrus DA, Dangl JL, Grant SR (2007) Global virulence regulation networks in phytopathogenic bacteria. Trends Microbiol 15: 363–371. 17627825 10.1016/j.tim.2007.06.005

