# Supplementary figures and images for: Community Structure and Functional Gene Profile of Bacteria on Healthy and Diseased Thalli of the Red Seaweed Delisea pulchra
Source: PLoS One. 2012 Dec 3;7(12):e50854. doi: 10.1371/journal.pone.0050854 (PMC3513314; doi:10.1371/journal.pone.0050854)

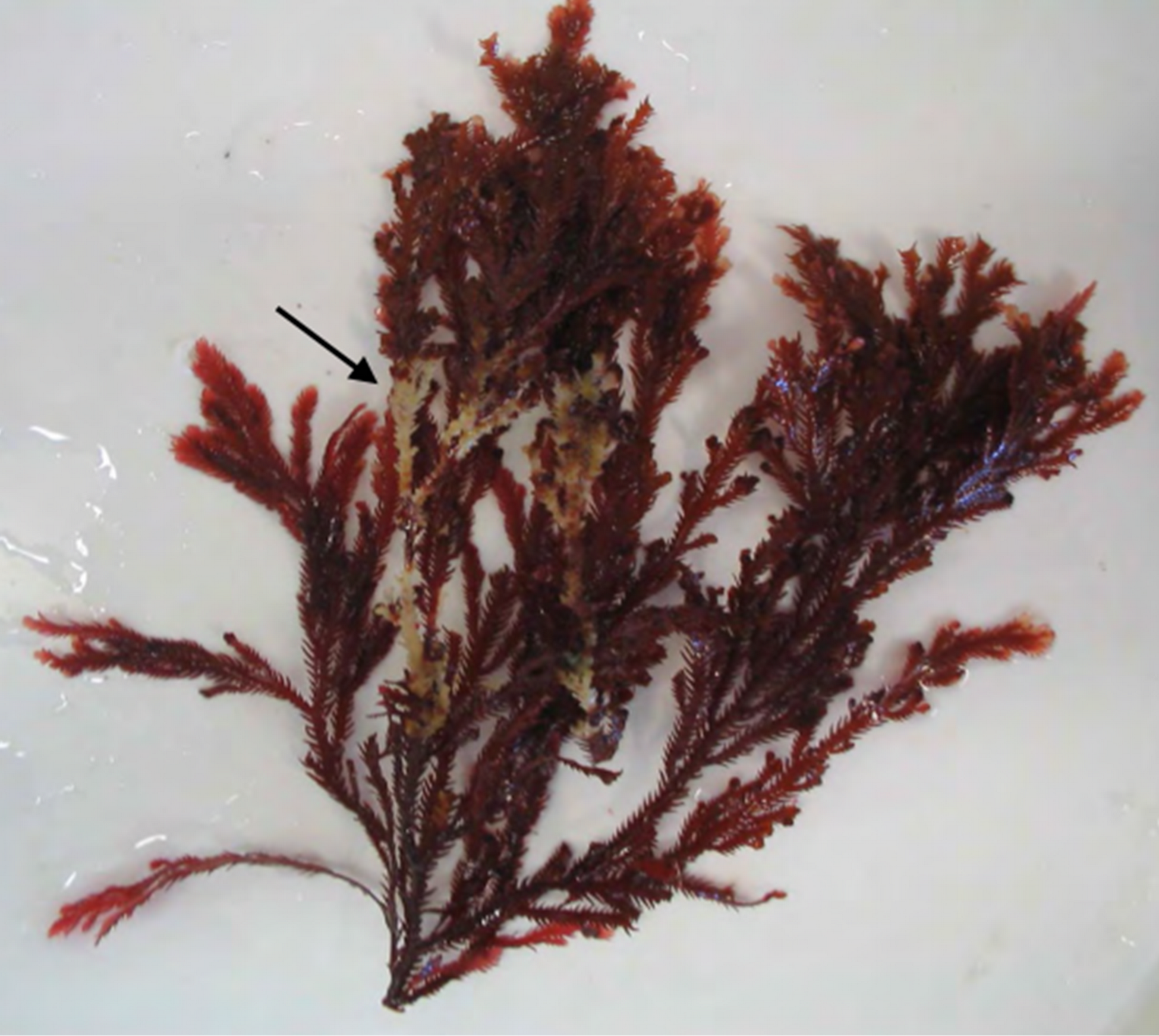

Supplement: Figure S1 — Bleached Delisea pulchra collected from Bare Island, Sydney, Australia. The bleached section in the mid thallus region is indicated by the arrowhead. (TIF) [file pone.0050854.s001.tif]

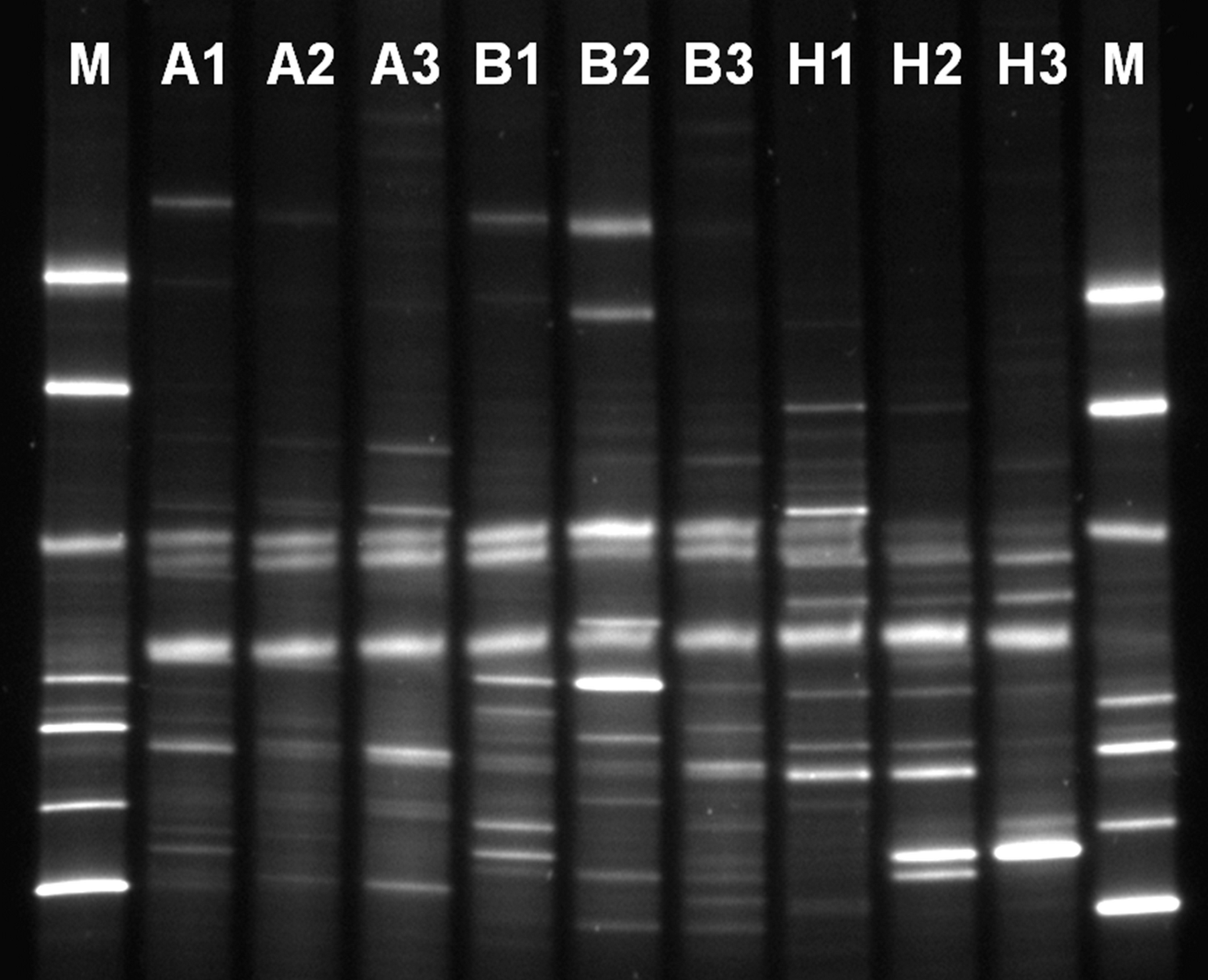

Supplement: Figure S2 — Comparison of different samples by DGGE fingerprinting. Lane M: DGGE Markers, Lanes A1, A2 and A3 contain DGGE bands from tissue adjacent to bleached tissue, Lanes B1, B2 and B3, DGGE bands from bleached tissue, Lanes H1, H2 and H3, DGGE bands from healthy tissue. (TIF) [file pone.0050854.s002.tif]

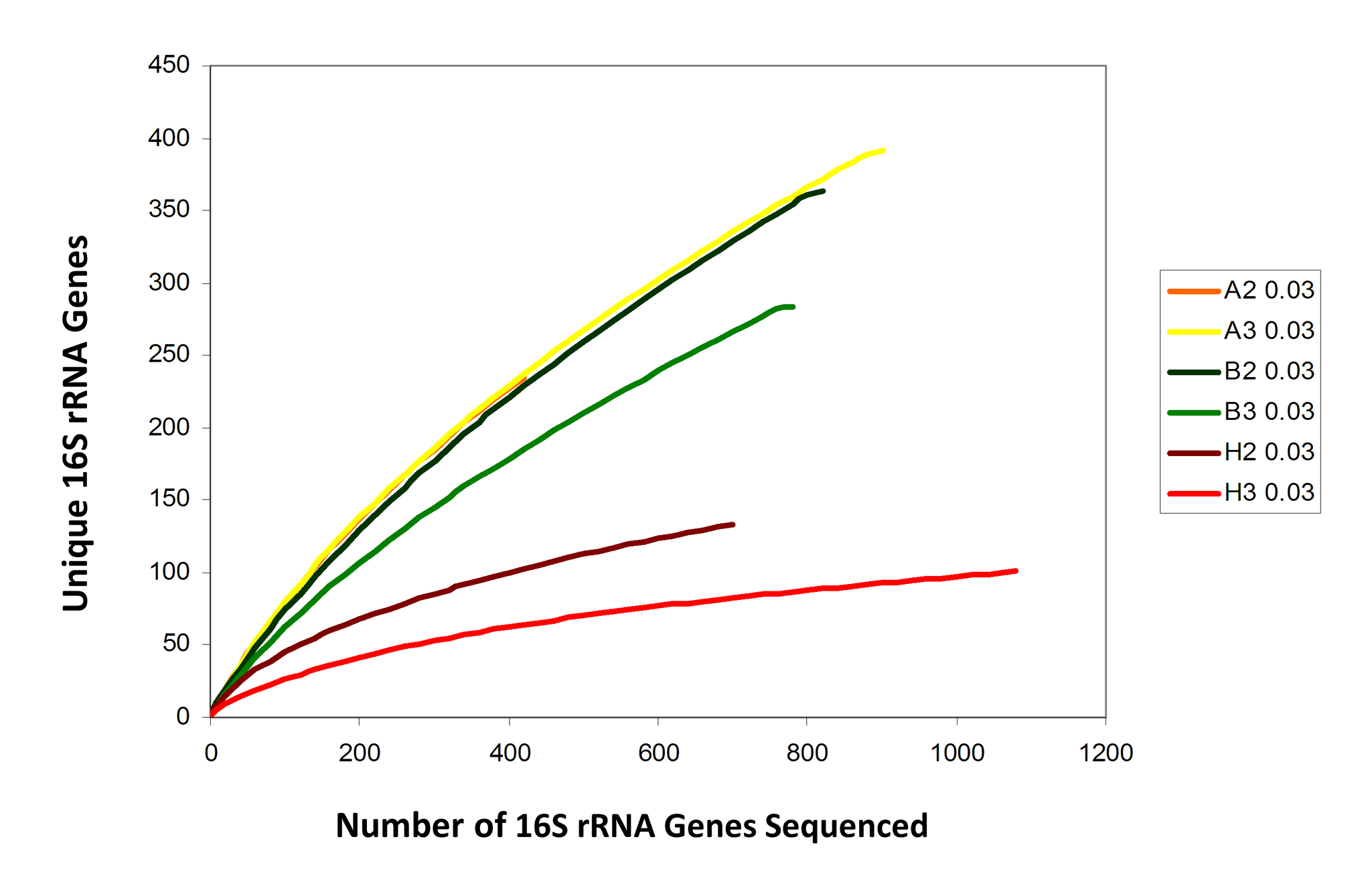

Supplement: Figure S3 — Rarefaction curves comparing the number of OTUs at a distance of 0.03 between 16S rRNA gene libraries constructed bleached tissue (B), from tissue adjacent to bleached tissue (A) and healthy tissue (H). (TIF) [file pone.0050854.s003.tif]

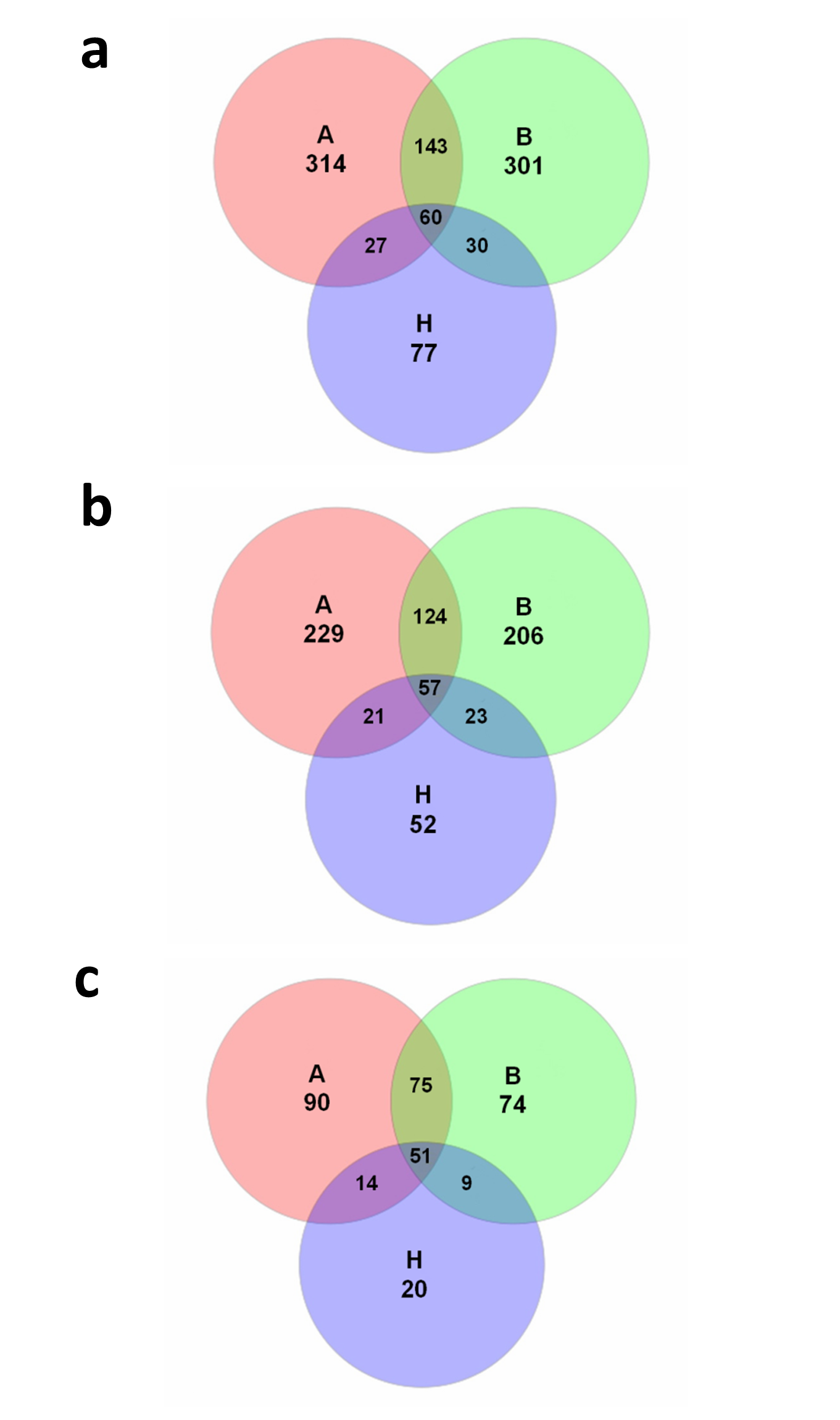

Supplement: Figure S4 — Venn diagrams showing the number of OTUs shared between communities on bleached tissue (B) healthy tissue (H) and adjacent tissue (A) with OTUs at 0.03 difference. (TIF) [file pone.0050854.s004.tif]

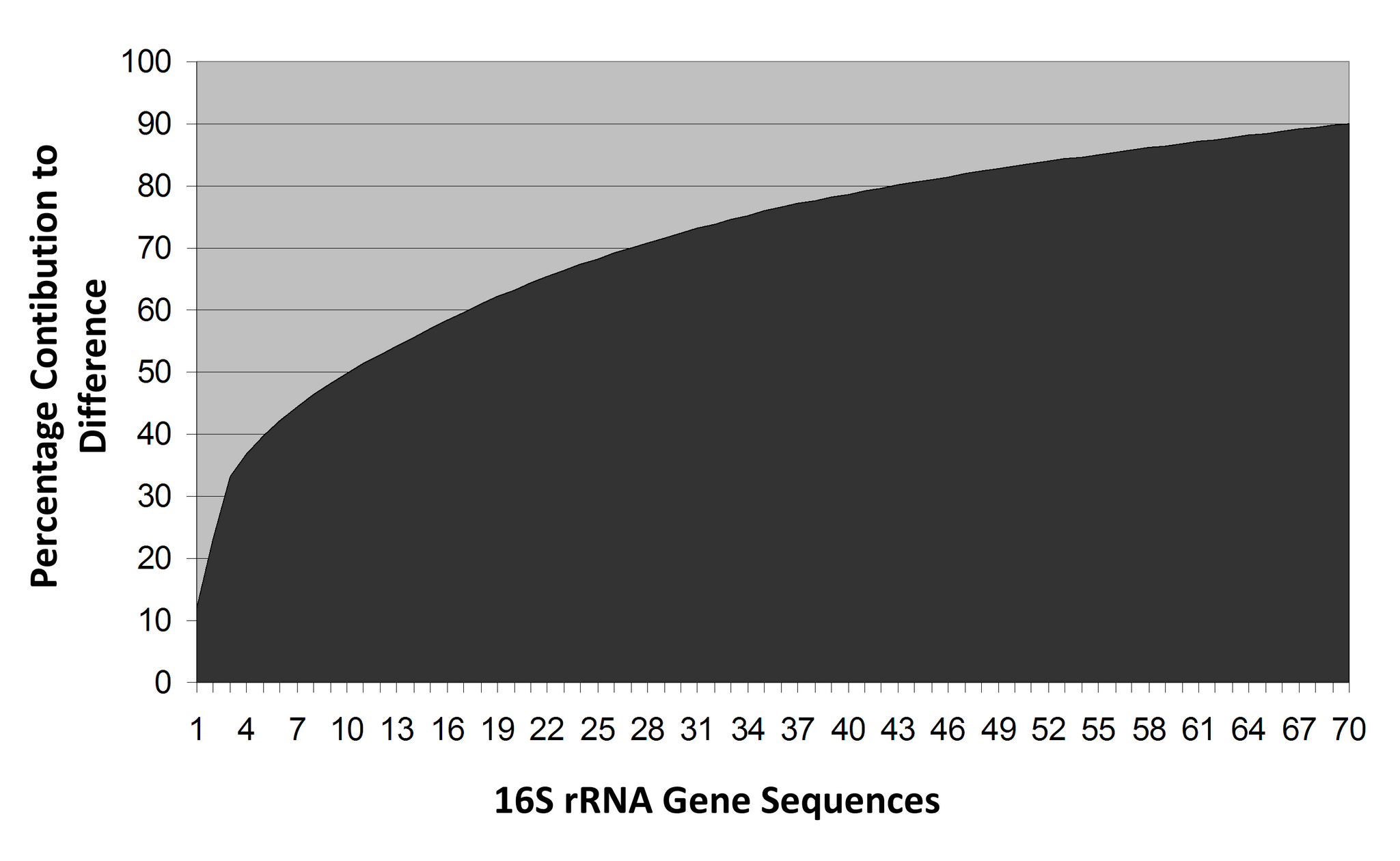

Supplement: Figure S5 — The contribution of different OTUs (at 0.03 sequence difference cut-off) to difference between 16S rRNA genes libraries from bleached and healthy samples. (TIF) [file pone.0050854.s005.tif]

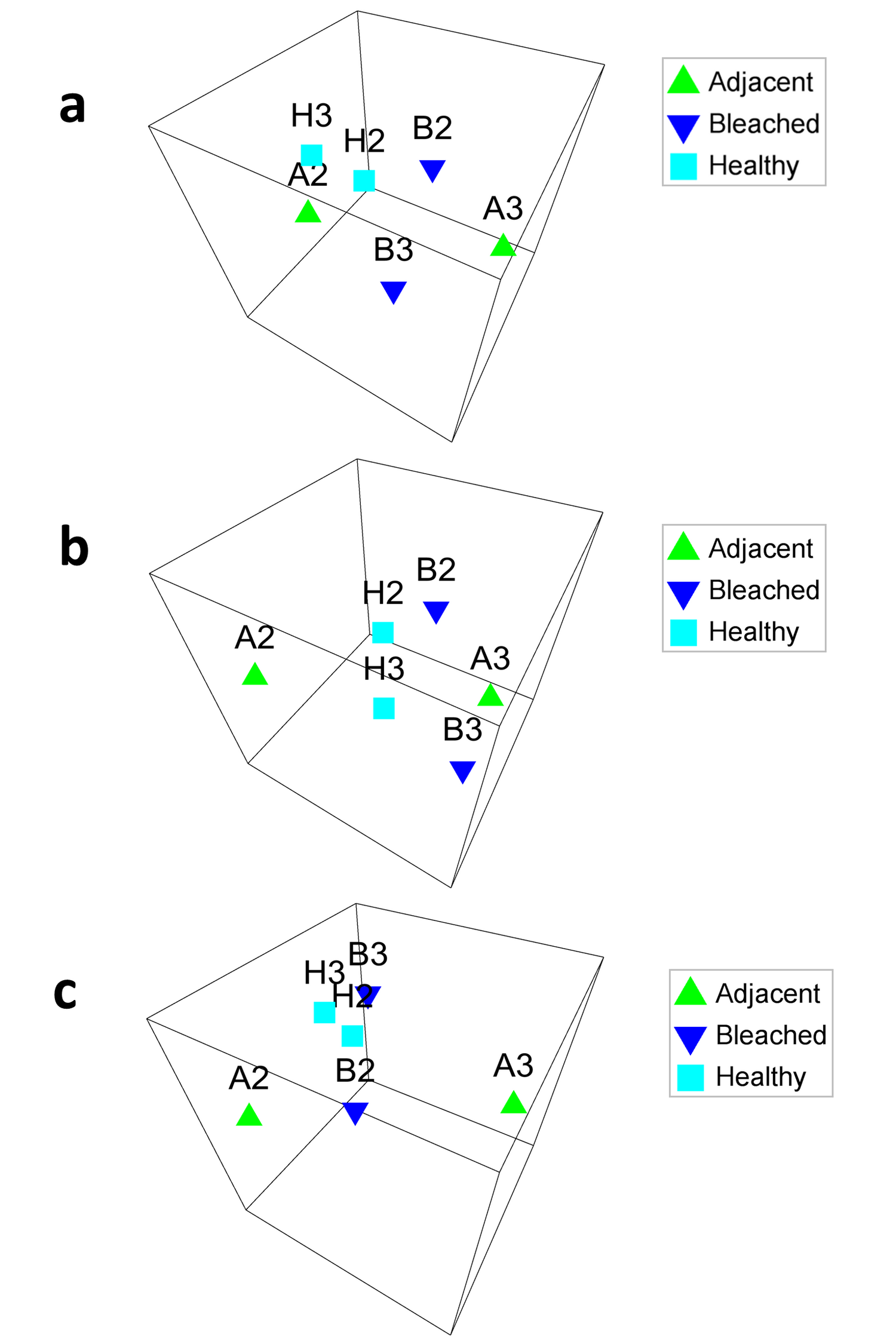

Supplement: Figure S6 — Multidimensional-scaling (MDS) plots comparing the level of similarity between metagenomic libraries constructed from bleached tissue (B), from tissue adjacent to bleached tissue (A) and healthy tissue (H) using a matrix containing ORFs that could be matched to COGs at E-value cut-offs smaller 10−5 (1), 10−10 (2) and 10−20 (3). (TIF) [file pone.0050854.s006.tif]

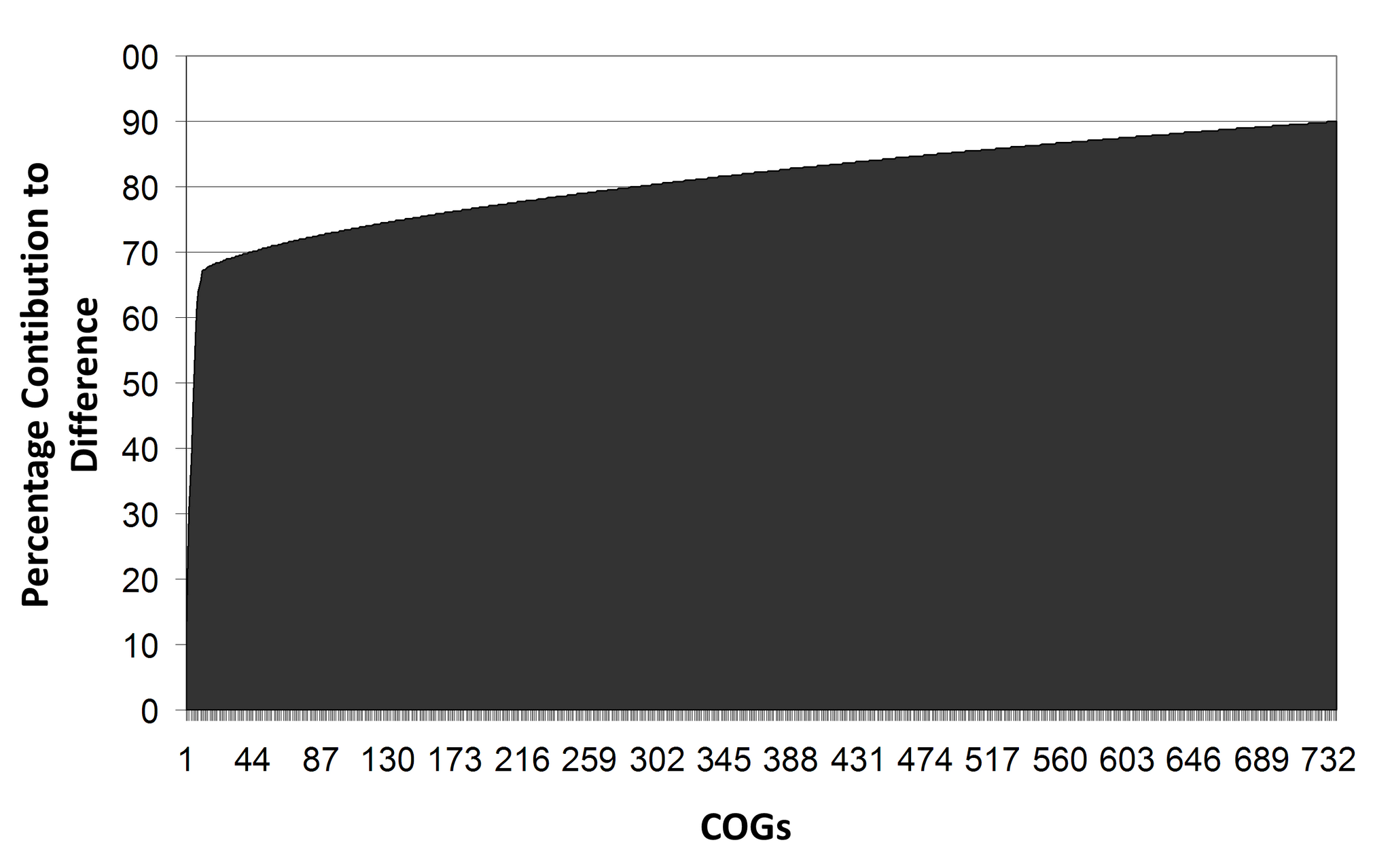

Supplement: Figure S7 — The contribution of individual COGs to the difference between metagenomic libraries from bleached and healthy samples. (TIF) [file pone.0050854.s007.tif]
