# Supplementary material for: Community Structure and Functional Gene Profile of Bacteria on Healthy and Diseased Thalli of the Red Seaweed Delisea pulchra
Source: PLoS One. 2012 Dec 3;7(12):e50854. doi: 10.1371/journal.pone.0050854 (PMC3513314; doi:10.1371/journal.pone.0050854)
Supplement: Text S1 — Supplementary Material and Methods. (DOC) [file pone.0050854.s008.doc]

**Supplementary Material and Methods:**

**Community DNA isolation**

Prior to extraction, the algal thallus (20 gm) was washed three times in 300 ml of sterile calcium- and magnesium-free sea water (CMFSW) containing 0.45 M NaCl, 10 mM KCl, 7 mM Na_2_SO_4_, 0.5 mM NaHCO_3_ and 10 mM EDTA, to remove loosely associated bacteria and invertebrates. Tissue samples (2.5 gm wet weight) were then individually excised from three samples of healthy and bleached tissues, as well as tissue adjacent to bleached tissue of the algal thallus from three individual bleached and healthy algal specimens. Excised tissue was added to 100 ml extraction buffer, which consisted of CMFSW along with filter-sterilized and 1:100 diluted Rapid Multi-Enzyme Cleaner (3M, NSW, Australia). The samples were incubated for two hours at room temperature on a shaker at 80 rpm. The supernatant from the extraction was filtered through an 11 µm filter, and the samples were extracted with an equal volume of phenol: chloroform: isoamyl alcohol (25:24:1) (Fluka, Seelze, Germany). Tubes were mixed by inversion and centrifuged at 10 000 x g for 10 minutes and the aqueous phase transferred to new tubes. The community DNA was precipitated with 0.3 M sodium acetate (pH 5.2) and 0.6 volumes isopropanol overnight and the DNA pellets were recovered by centrifugation at 20 000 x g and 4^0^C for 30 minutes. The DNA pellets were washed with 70% ethanol, air-dried and re-suspended in a total of 500 μl of sterile deionised water, after which samples were stored in the -80^0^C freezer. The yield and quality of the extracted DNA was verified by agarose gel electrophoresis.

**Community fingerprinting by denaturing gradient gel electrophoresis**

A nested PCR-denaturing gradient gel electrophoresis (DGGE) approach was used to obtain a community fingerprint of the surface-associated bacterial community . The 16S rRNA gene from each algal community DNA extract was ampliﬁed by the universal 16S rRNA gene primer pair 27F-1492R under the PCR conditions described above. Amplicons from the first round of PCR were diluted 1:100 with sterile, molecular-grade water and amplified using the bacterial primers 338F (ACT CCT ACG GGA GGC AGC), containing a 40 bp GC clamp and 530R (C CGC CCG CCG CGC CCC CGC CCC GGC CCG CCG CCC CCG CCC ACT CCT ACG GGA GGC AGC) targeting the V3 variable region of the 16S rRNA gene. Each 50 µl PCR reaction mixture contained 200 µM of each dNTP, 0.4 µmole of each primer, 0.1 mg/ml BSA, 1.0 U of *Taq* DNA polymerase, and 1X reaction buffer along with 1.5 mM MgCl_2_. The PCR program consisted of initial denaturation at 94°C for 3 min followed, by 30 cycles of amplification. Each amplification cycle consisted of denaturation of the target genes at 94°C for 30 seconds, annealing at 57°C for one min, and extension at 72°C for 1.5 min. A 10% polyacrylamide gel and 35% to 55% denaturing gradient of 7 M urea plus 40% (vol/vol) formamide was used for electrophoresis in 1X TAE buffer at 60 °C, for 16 h at 75 V using a DCode universal mutation detection system (Bio-Rad Laboratories, Hercules, Ca, USA). After electrophoresis, gels were stained with 1X SYBR Gold (Invitrogen) for 30 minutes in 1X TBE buffer and visualized under a UV trans-illuminator. DGGE bands were then digitalized and analyzed using Quantity One gel analysis software (Bio-Rad Laboratories, Hercules, Ca, USA). Band analysis was performed using the rolling disk method and setting background subtraction at 15. Bands with intensity <0.05 were excluded from the analysis. Unweighted pair-group methods with arithmetic mean (UPGMA) trees were generated in Quantity One, using Sorensen's similarity index.

**Annotation of metagenomic sequences**

Annotation of metagenomic sequences was based on previously established procedures . In order to filter out eukaryotic DNA contamination, arising from co-extraction of algal DNA, the assembled data was filtered by searching all DNA fragments against The National Center for Biotechnology Information (NCBI) nucleotide database (NT) using BLASTN . The Megan algorithm was then used to classify sequences from the BLAST search into taxonomic groups with parameters “min score” set at 30 and “top score” set at 10% . Manual evaluation confirmed that this procedure effectively removed scaffolds and singletons derived from eukaryotic DNA. All ORFs associated with putative eukaryotic DNA fragments were omitted from the functional comparison. Open reading frames (ORFs) were identified from scaffolds and singletons with Metagene , a program that is specific for prokaryotic gene detection from environmental genome shotgun sequences. For functional annotation, each ORF was searched against the COG databases using HMMER version 2.3.2 and the curated section of SwissProt by means of BLASTP, with a confidence cut-off of 10^-20^. In order to estimate the abundance of each functional assignment, the functional annotation of each ORF was multiplied by the average coverage of the DNA fragment, as described by Thomas et al. (2010).

**References:**

1. Muyzer G, Smalla K (1998) Application of denaturing gradient gel electrophoresis (DGGE) and temperature gradient gel electrophoresis (TGGE) in microbial ecology. Antonie van Leeuwenhoek 73: 127-141.

2. Lane D (1991) 16S/23S rRNA sequencing. Nucleic Acid Techniques in Bacterial Systematics (Stackebrandt E & Goodfellow M, eds). J Wiley & Sons, Chichester: 115–175.

3. Thomas T, Rusch D, DeMaere MZ, Yung PY, Lewis M, et al. (2010) Functional genomic signatures of sponge bacteria reveal unique and shared features of symbiosis. The ISME Journal 4: 1557-1567.

4. Altschul SF, Madden TL, Schaffer AA, Zhang J, Zhang Z, et al. (1997) Gapped BLAST and PSI-BLAST: a new generation of protein database search programs. Nucleic Acids Research 25: 3389-3402.

5. Huson DH, Auch AF, Qi J, Schuster SC (2007) MEGAN analysis of metagenomic data. Genome Research 17: 377.

6. Noguchi H, Park J, Takagi T (2006) MetaGene: prokaryotic gene finding from environmental genome shotgun sequences. Nucleic Acids Research 34: 5623-5630.

7. Tatusov RL, Fedorova ND, Jackson JD, Jacobs AR, Kiryutin B, et al. (2003) The COG database: an updated version includes eukaryotes. BMC Bioinformatics 4: 41-54.

8. Eddy SR (1998) Profile hidden Markov models. Bioinformatics 14: 755-763.

9. Boeckmann B, Bairoch A, Apweiler R, Blatter MC, Estreicher A, et al. (2003) The SWISS-PROT protein knowledgebase and its supplement TrEMBL in 2003. Nucleic Acids Research 31: 365-370.

**Figure Legends:**

**Figure S1:** Bleached *Delisea pulchra* collected from Bare Island, Sydney, Australia. The bleached section in the mid thallus region is indicated by the arrowhead.

**Figure S2:** Comparison of different samples by DGGE fingerprinting. Lane M: DGGE Markers, Lanes A1, A2 and A3 contain DGGE bands from tissue adjacent to bleached tissue, Lanes B1, B2 and B3, DGGE bands from bleached tissue, Lanes H1, H2 and H3, DGGE bands from healthy tissue.

**Figure S3:** Rarefaction curves comparing the number of OTUs at a distance of 0.03 between 16S rRNA gene libraries constructed bleached tissue (B), from tissue adjacent to bleached tissue (A) and healthy tissue (H).

**Figure S4:** Venn diagrams showing the number of OTUs shared between communities on bleached tissue (B) healthy tissue (H) and adjacent tissue (A) with OTUs at 0.03 difference.

**Figure S5:** The contribution of different OTUs (at 0.03 sequence difference cut-off) to difference between 16S rRNA genes libraries from bleached and healthy samples

**Figure S6:** Multidimensional-scaling (MDS) plots comparing the level of similarity between metagenomic libraries constructed from bleached tissue (B), from tissue adjacent to bleached tissue (A) and healthy tissue (H) using a matrix containing ORFs that could be matched to COGs at E-value cut-offs smaller 10^-5^ (**1**), 10^-10^ (**2**) and 10^-20^ (**3**).

**Figure S7:** The contribution of individual COGs to the difference between metagenomic libraries from bleached and healthy samples.
